# Resilience and Experience of the COVID-19 Pandemic among Italian University Students: A Mixed-Method Study

**DOI:** 10.3390/ijerph191811714

**Published:** 2022-09-16

**Authors:** Firas Mourad, Sonia Mangialavori, Antonella Delle Fave

**Affiliations:** 1 Department of Physiotherapy, Exercise and Sports, LUNEX International University of Health, 4671 Differdange, Luxembourg; 2 Luxembourg Health & Sport Sciences Research Institute A.s.b.l., 50, Avenue du Parc des Sports, 4671 Differdange, Luxembourg; 3 Department of Pathophysiology and Transplantation, Università degli Studi di Milano, Via Francesco Sforza 35, 20122 Milan, Italy

**Keywords:** resilience, subjective experience, university students, COVID-19 pandemic, mixed-method approach

## Abstract

A vast amount of literature has highlighted that restrictions imposed by the COVID-19 pandemic, such as lockdowns and the resulting interruption of face-to-face academic activities, strongly disrupted students’ daily routine and undermined their well-being. Through a mixed method approach, this study was aimed at investigating the association between students’ experience of the health emergency and their resilience levels during the first pandemic outbreak. Between April and May 2020, 421 Italian university students attending Health Sciences, Humanities, and Political Sciences courses completed the Resilience Scale for Adults (RSA), provided narratives about the emergency by answering an open-ended question, and filled out a demographic questionnaire. Results showed that narratives about community/society issues were by far the most recurrent ones across disciplinary areas, while a significantly higher percentage of students from Humanities focused on study/university. Health Sciences students were more likely to provide narratives concerning social commitment, and they reported significantly higher resilience levels than Humanities students. A higher percentage of students with moderate resilience focused their narratives on the study/university domain, compared to students with high resilience. Findings suggest the importance of supporting students’ resilience to counterbalance their academic concerns in both times of crisis and ordinary times.

## 1. Introduction

On 11 March 2020, the World Health Organization declared a global pandemic due to the SARS-CoV-2 coronavirus [1]. The rapid evolution of the emergency severely impacted the lives of individuals and communities, with negative consequences at the political, health, financial and social levels. In the attempt to limit the contagion spread, governments adopted protective and preventative measures such as general lockdowns and curfews lasting for long periods, which negatively impacted all productive sectors of the society [2]. Such a complex scenario, associated with uncertainty and fear, had negative repercussions on individuals’ mental health. An increased prevalence of affective disorders and suicide rates was detected in the general population, among health professionals directly engaged in the treatment of infected patients, and among persons with pre-existing psychological disorders [3,4,5,6,7]. University students—previously identified as specifically exposed to academic and social stressors, with their negative consequences on mental health—during the pandemic faced remarkable uncertainty in a variety of domains, such as academic achievements, job perspectives, personal and family life projects [8]. Concerns on mental health implications of the pandemic were raised by the scientific community, as the long-term repercussions are not fully known yet [9].

### 1.1. Resilience as a Resource to Face Adversities

Psychological resilience is broadly defined as the preservation of good mental health despite the exposure to adversity, by virtue of the mobilization of personal and relational resources to overcome difficulties [10,11]. The conceptualization of resilience was recently challenged and shifted from considering it as a process of “bouncing back” to previous psychosocial conditions after facing an adverse event, to a developmental process of “bouncing forward” or “building back better”, reinforcing and acquiring new resources through learning and change. Generally, resilience is conceptualized in three ways: a dynamic process, a more stable trait, and an outcome in response to adversity [10,11]. The most recent advancements in this domain converge towards a definition of resilience as a complex dynamic process resulting from the interaction between individual factors (genetic and epigenetic characteristics) and socioenvironmental factors (such as family and social support, community networks, public services), which allow to preserve mental health and to grow amidst adversities. Notably, resilience not only includes the individual strategies adopted in the face of significant risk factors, but also personal and environmental protective factors that promote a positive adaptation. 

To assess resilience as a multidimensional process, specific measurement instruments were designed to investigate three core components: individual dispositional traits; family climate and cohesion; and support from external systems that reinforces coping strategies [12,13]. One of the most comprehensive research tools currently available to assess resilience from a processual perspective is the Resilience Scale for Adults (RSA), developed by Friborg et al. (2003) with the aim of assessing protective personal and environmental factors which may contribute to a positive adaptation to adversities [14,15].

A meta-analysis confirmed that resilience is a psychological resource that can support the acceleration of recovery and the mitigation of the negative effects of a crisis [16]. Moreover, recent studies conducted during the COVID-19 pandemic have identified the protective role of resilience in distress conditions among several populations, including university students [7,17,18,19]. 

Considering the manifold implications of the pandemic for citizens’ daily life at the psychological, relational, and community levels, and the resources that were mobilized by individuals and groups to cope with the related challenges at each of these levels, the present study relies on the dynamic view of resilience, as a multidimensional process involving both individual and environmental components [14,15]

### 1.2. Positive and Negative Dimensions of Students’ Experience during the Pandemic 

The health emergency due to the pandemic has placed an unprecedented overload on students, a population showing high distress levels in previous studies, and affected their ability to rely on ordinary coping strategies [20]. Comparing students’ psychophysical conditions and behaviour before and during the pandemic, Huckins et al. (2020) detected a significant increase in sedentary lifestyle, depression, and anxiety in relation to the changes and restrictions occurring at the university and societal levels to circumscribe the infection spreading [21]. Using a mixed-method approach, Browning et al. (2021) examined the psychological impact of COVID-19 among students of United States public universities, highlighting that women and students spending eight hours or more daily on screens reported poorer general health status [22]. Further, when multiple risk factors were investigated together, students aged 18 to 24 years and with acquaintance infected by SARS-CoV-2 were more likely to experience higher psychological distress. High levels of anxiety regarding participants’ social life, academic achievements, and future job career emerged as a major risk factor in the study [22]. These findings are in line with other ones obtained across countries during the pandemic: in China, 25% of the university students showed symptoms of anxiety [23]; in the United States, findings obtained from college students highlighted increased anxiety in 38.5% of the participants, stress in 71%, and depression in 38.5% [24]; in Spain, students attending courses in Humanities, Social Work, and Law experienced moderate to severe anxiety [25].

Comparative studies investigating the differences in psychological distress levels reported by students attending different university courses were conducted over the years, detecting higher rates of depression and anxiety and a more frequent use of psychotropic substances among medical students [26,27,28]. During the pandemic, a different picture emerged, with Health Sciences students reporting lower levels of affective disorder symptoms, compared to students attending other courses [29,30,31,32]. 

One of the first Italian surveys exploring the experience and behaviours of undergraduates during the first wave of the pandemic (the EPICO study) did not detect relevant changes in health-related behaviours (i.e., diet and smoking), while most participants reported reduced physical activity. Most students, especially those attending Life Sciences courses, showed a good level of knowledge and understanding of the health emergency and related preventive measures [33]. Conversely, another study involving Italian undergraduate students [34] identified anxiety and depression symptoms in higher percentages (35% and 73% respectively) of participants. The increased risk for anxiety was mainly related to being female, being forced to attend university remotely, and not being able to meet one’s partner. Finally, Villani et al. [34] found that physical activity was a protective factor for mental health during the pandemic. These findings can be related to the peculiar situation in Italy, the first and most affected country in Europe during the first COVID-19 outbreak. In order to contain the contagion spread, the government imposed restrictions and a prolonged lockdown, during which people could not leave their homes except for basic needs; the 71 days of total lockdown taking place in Spring 2020 [23] and the closure of schools and universities for a longer period than in other countries [34,35] may have further contributed to the distress of Italian students. 

Researchers have also investigated the positive dimensions of students’ experience during the pandemic, such as perceived institutional and family support, teachers’ empathy, and economic resources [23,36]. To date, only one Italian study has investigated the protective role of resilience on university students’ adjustment to the pandemic, finding a negative relationship of resilience with perceived stress, study fatigue, and difficulties in learning and interpersonal relationships [17]. 

### 1.3. Aims of the Study

Based on the aforementioned literature, and considering the dearth of research jointly exploring students’ personal resources and qualitative narratives about the pandemic, this study was primarily aimed at jointly investigating, through a mixed-method approach, evaluations of the emergency condition and resilience levels among students of the University of Milano, during the first wave of the COVID-19 pandemic. Because little is known about the experience of the pandemic reported by students from different disciplines and considering that the specialized theoretical and practical knowledge students in Health Sciences were acquiring on topics related to healthcare and disease treatment—core aspects of the pandemic crisis—participants were divided into three groups, according to their area of study: Health Sciences, Humanities, and Political Sciences. Some differences concerning salient aspects of the pandemic reported in the qualitative narratives were hypothesized to emerge on the basis of participants’ disciplinary perspective. The second aim of the study was to compare groups as concerns resilience level and narratives about the pandemic, hypothesizing a relationship between the degree of resilience and the students’ views of the emergency. In particular, participants with higher levels of resilience were expected to report more constructive and resource-focused narratives of the situation.

To the best of our knowledge, this is the first study adopting a mixed-method approach to analyse the experience of university students during the COVID-19 lockdown period in relation to their resilience levels and across different university curricula.

## 2. Methods 

### 2.1. Study Design

An online cross-sectional survey was conducted among students at the University of Milano. Qualitative data were collected through optional open-ended questions, and quantitative ones through a set of scaled questionnaires. The adoption of a mixed-method approach was grounded in researchers’ interest to jointly explore university students’ well-being levels as well as personal views and experiences during the lockdown period characterizing the first wave of the pandemic. 

### 2.2. Participants

Data were collected through an online survey among 1799 undergraduate and postgraduate students from the University of Milano, attending courses in different disciplinary areas. A total of 421 participants were eligible for this study, as they had provided answers to an optional question regarding their view of COVID-19 emergency. Participants were divided into three groups depending on their study area: 294 students (Mage = 23.64, sd = 5.31) attended courses in Health Sciences, 68 (Mage = 23.43, sd = 5.35) in Humanities, and 59 (Mage = 24.47, sd = 8.13) in Political Sciences. Table 1 summarizes the demographic characteristics of the three groups. No significant differences were detected in this regard: each group was composed predominantly of females, full-time undergraduate students, unmarried and living with their family of origin.

### 2.3. Research Instruments

To the purpose of this study, participants’ demographic features, resilience levels, and free narratives on the pandemic situation elicited by an optional open-ended question were taken into account. Resilience was assessed through the validated Italian version of the *Resilience Scale for Adults* (RSA) [14,37]. RSA is a multidimensional scale consisting of 33 items grouped in six subscales: four subscales refer to individual resources (positive self-perception, positive perception of the future, social competences, structured lifestyle); one subscale assesses to family resources (with items referring to cohesion and mutual support); the last subscale refers to social resources (in terms of extra-family relationships and community support) [14]. Higher RSA scores indicate better adaptation to psychosocial adversities. The RSA proved to be a valid tool, as higher scores are related to a significant difference between healthy controls and psychiatric patients [14], a well-adjusted personality profile [14], a higher tolerance to pain, and a decreased negative impact of stressful life events on psychological well-being [38]. For the total RSA score, in this study, alpha coefficient was 0.85.

Participants’ experience of the COVID-19 emergency was explored through the following optional open-ended question “Do you have any supplementary comments regarding the COVID-19 emergency?”.

### 2.4. Procedures

The study was first submitted for approval to the Ethics Committee of the University of Milano. The technological office staff of the University uploaded the questionnaires on the institutional digital platform, making the survey accessible for online participation. Upon agreement with the presidents of a variety of bachelor and master courses, a web-link to the survey was distributed to students via the institutional mailing list; interested students could fill out the survey between 15 April and 15 May 2020, during the first lockdown period imposed by the Italian government to cope with the health emergency. After reading the introduction and the objectives of the survey, participants were given the opportunity to ask for clarifications via email. Providing an online informed consent was mandatory to access the survey questions. The survey took 15 to 30 min to complete, depending on the students’ decision to answer the optional open-ended questions, located at the end of the questionnaire package. Participation was voluntary, and students were free to withdraw at any time while filling out the survey. The data provided by the participants who decided to complete the survey and submit it were digitally stored; anonymity was preserved in all phases of the data collection. Given the expected participation of a large number of students, a priori calculation of the sample size to determine the statistical power for quantitative measures was not deemed as necessary.

### 2.5. Data Processing and Analysis

The data were transferred and stored on an encrypted computer for the sole purpose of analysis. Access was granted only to researchers involved in the study. No hard copy or sensitive data were collected. All data were organized in Microsoft Excel 2020 tables. Subsequently, they were encoded and processed with the SAS^®^ statistical package (SAS. Version 9.4 for Windows; SAS Institute Inc., Cary, NC, USA). Analyses of categorical data included calculation of frequencies and percentage distributions, as well as exploration of their differences across participant groups through the Chi-square procedure. Quantitative data were analysed with descriptive procedures and parametric inferential techniques.

### 2.6. Coding Procedure of Qualitative Data

The answers to the open-ended question required an accurate coding process [39]. Given their complexity and articulation, they often had to be portioned into multiple semantic units. A numerical code was assigned to each semantic unit. For each participant, up to 4 units were coded and retained for analyses. Every semantic unit was classified within broader categories, following a hierarchical system. This coding procedure is based on the bottom-up approach typical of quality of life studies conducted within the psychological and socio-contextual domains [40]. The whole encoding procedure was performed by two trained raters, to ensure the reproducibility and trustworthiness of the process: a trained author extracted the semantic units, grouped them into broader categories, and then encoded both units and categories; a second author, expert in the field and in this methodology, reviewed each phase of the process providing feedback and suggestions. Any discrepancies were discussed until a consensus was reached, and in some cases, a third expert rater was involved [41]. This process was applied to each of the answer units, leading to a final codebook comprising 12 general answer categories: work, study/university, social commitment, community/society issues, health management, media/information, family relations, interpersonal relationships, physical health, negative thoughts/emotions, positive thoughts/emotions, and other. A total of 778 semantic units were collected and categorized.

### 2.7. Statistical Analyses

The analysis of the qualitative data was aimed at exploring the contents of students’ personal experience, and at comparing them across the three disciplinary areas of attendance. Since most students provided multiple answer units, the number of participants mentioning each answer category in at least one unit was calculated. It was therefore possible to compare the percentage of participants providing answers in each category across the three disciplinary groups, by calculating χ^2^ on 2 × 3 frequency tables.

As concerns analysis of the quantitative data, descriptive statistics were first calculated for RSA. To identify differences between the three participant groups in the total resilience score, a univariate ANOVA with Tukey’s Studentized Range post hoc comparison was run. In addition, the distribution of participants within three resilience levels (i.e., low, moderate, and high) was calculated. A comparison was then performed between the percentage of participants mentioning each qualitative category, based on their resilience level by calculating χ^2^ on 2 × 2 frequency tables.

The significance level was set at *p* value < 0.05 for all comparisons.

## 3. Results

The qualitative and quantitative findings are presented separately for each disciplinary group to allow for comparisons.

### 3.1. Qualitative Findings

Table 2 provides the frequency and percentage distribution of the qualitative answer units across categories, separately for the three disciplinary areas. Table 3 provides a more nuanced description of the contents within the major answer categories, namely the categories including at least 5% of the answer units across the three groups of participants. We analyse these contents more closely.

In the answers referring to study/university, participants primarily emphasized the difficulties faced in adapting to remote learning activities and the institutional problems in establishing online teaching and training procedures. These critical aspects were especially emphasized by students of Health Sciences, who could not benefit from active participation in clinical trainings and face-to-face educational activities, essential to acquire technical competences and skills. Participants in the other two groups, instead, who attended courses characterized by more theoretical and less specialized technical contents, and by a greater freedom granted to students in building individualized learning pathways, provided a more balanced distribution of negative and positive answer contents, the latter highlighting the positive sides of remote learning, such as higher flexibility in organizing the individual study schedule, and an adequate management of online activities by the university.

In the domain of community/ society issues, answers referred to present and future challenges and opportunities, revolving around two main themes. The first one concerned national and local governance strategies in coping with the emergency; at the negative level, answers emphasized organizational failures, unequal distribution of resources, and lack of attention to the frailest sectors of the society; on the positive side, they were focused on the opportunities provided by the pandemic crisis to implement more adequate health, social and ecological policies. The second theme was represented by comments on citizens’ behaviours, highlighting negative ones such as selfishness and neglect of the restriction rules, as well as positive ones such as solidarity and responsibility. The evaluation of the COVID-19 pandemic as a collective, and the positive opportunity to share, learn, and grow as a community, was primarily underscored by students of the health domain, who could obtain a deeper and more direct knowledge of the relentless efforts made by health professionals and local stakeholders in trying to cope with and overcome problems and challenges.

*Negative thoughts/emotions* included a variety of feelings, prominently described through generic terms such as bad mood, negative emotions, malaise, and intolerance for freedom restrictions; feelings of stress, anxiety and uncertainty in the short term represented a more homogeneous subcategory of answers across the three groups of participants. A peculiar subcategory (though accounting for a limited number of answer units) emerged for Humanities students, who more frequently expressed a pessimistic view of the future. This result was possibly related to their difficulties in identifying their future role in the society, due to the broader and at the same time less specific contents of their curriculum, opening a variety of possible professional pathways and job opportunities that could generate access uncertainties related to the economic and social consequences of the pandemic.

As concerns *positive thoughts/emotions*, across the three groups of participants, the experience of the pandemic and related restrictions was primarily reported as an opportunity for personal growth, allowing for the rediscovery of values, personal strengths and potentials, including the availability of more time to engage in personal interests and to experiment new ones; hope for a conclusive solution of the pandemic was also often reported, followed by feelings of competence and mastery in adaptively coping with the challenges imposed by the critical situation.

### 3.2. Participant Distribution across Answer Categories by Study Area

The percentage of participants who provided at least one mention for each answer category across disciplinary areas is detailed in Table 4. The comparison of participants’ distribution across groups was investigated through the Chi-square procedure. For each of the 12 categories, a contingency 2 × 3 table was created (two answer options: 0 = no answer; 1 = at least one answer unit in the category; three disciplinary groups).

Across disciplines, participants primarily focused on *community/society* issues; however, a significantly lower percentage of students from the Health Sciences provided answers in this category, compared with the other two groups (*p* = 0.0448). The category *study/university* was mentioned by a significantly higher percentage of participants attending courses in the Humanities (*p* = 0.0217). As concerns *social commitment,* students in Health Sciences answered in significantly higher percentage than students from Humanities and Political Sciences (*p* = 0.0450); the calculated Chi-square must be however interpreted with caution, as only one participant for each of the other study areas mentioned this category. No group differences were found in the percentage of participants providing answers in the remaining categories.

### 3.3. Quantitative Findings

Following the approach adopted in previous studies [42,43,44], three levels of resilience were identified, dividing by three the maximum total scale score (=231). A low level of resilience corresponded to scores between 33 and 77; a moderate level of resilience was identified for scores between 78 and 154; and a high level of resilience corresponded to scores above 154. Table 5 illustrates, for each study area, the percentage distribution of participants across resilience levels and the RSA mean score. Although close to the cut-off value of 77, only one student from the Health Sciences showed a low level of resilience; this participant was thus excluded from the subsequent analysis. A higher percentage of students in the Health Sciences (70.68%) reported high levels of resilience compared to participants in the other groups, who were more equally distributed between moderate and high resilience levels (52.95% and 47.05% for students in Humanities, and 47.46% and 52.54% for students in Political Sciences, respectively). Moreover, the three groups significantly differed in the total resilience mean score (F = 11.23; *p* < 0.0001); the Tukey’s correction revealed a significant difference between the Health Sciences and Humanities groups (*p* < 0.05), while the Political Sciences group scored in between, not differing significantly from any of the others.

### 3.4. Association between Participants’ Resilience Levels and Mention of Qualitative Answer Categories

The association between participants’ level of resilience and their mentioning of each answer category was investigated through the Chi-square procedure. For each answer category, a contingency 2 × 2 table was created (two resilience levels: moderate and high; two answer classifications: 0 = no answer; 1 = at least one answer unit in the category).

As reported in Table 6, participants’ mentioning of the *study/university* category was differentially associated with levels of resilience (*p* = 0.0091). More specifically, the percentage of participants with moderate levels of resilience who mentioned this category in their qualitative answers was almost double than the percentage of participants with high resilience levels who did so. Moreover, when referring to the category *study/university,* the majority of participants with moderate levels of resilience focused on negative aspects, such as uncertainty about the academic future, difficulties in handling group works remotely, and negative consequences of online education. No other significant association was found across the other categories.

## 4. Discussion

To the best of our knowledge, this is the first mixed-method study aimed at investigating the experience of university students during the COVID-19 pandemic, through the exploration of the association between their levels of resilience and their experience of the health emergency. At the conceptual level, resilience refers to a multidimensional, dynamic process allowing an individual to thrive and preserve well-being under adversarial conditions; it is therefore interesting to investigate it in association with subjective narratives during the first phase of the pandemic, especially in a country such as Italy which was massively hit by the disease outbreak. A strict national lockdown disrupted citizens’ daily routine, and the still limited knowledge about SARS-CoV-2 infection, clinical manifestation and treatment options generated deep uncertainty and distress in all sectors of the population. At the empirical level, findings were collected and compared across three demographically homogeneous groups of participants, and the adoption of a mixed-method approach gave participants the opportunity to freely describe their personal feelings, experiences, emotions, and thoughts regarding the health emergency.

### 4.1. Students’ Experience of the Pandemic Emergency

The qualitative information collected in this study shed light on the personal experiences of university students, contextualized into an extraordinary event such as the COVID-19 pandemic. Across disciplinary areas, the participants mainly focused on few topics and domains. Narratives concerning the collective dimensions of the emergency, grouped in the category *community/society issues,* were by far the most frequent ones, followed by considerations on the effects of the pandemic on academic activities and study schedule, and by personal life reflections and feelings. Across groups, less attention was paid to relational aspects involving family and friends, work, and emergency health management. These group similarities in answer pattern can be partly referred to the homogeneous demographic features of the participants; in addition, they shed light on some globally perceived consequences of the pandemic, at least among college students.

Researchers have highlighted that being a university student during the lockdown was a risk factor for experiencing loneliness (the feeling that one’s social needs are not quantitatively or qualitatively satisfied by one’s social relationships), and related negative implications [45,46,47]. Active and adaptive coping strategies emerged as effective resources for counterbalancing loneliness and its consequences [48,49]. Other studies conducted during the pandemic suggested that at the relational level social distancing [50] may induce supportive coping mechanisms [23]. The increased use of internet and social media taking place during the lockdown emerged as the most common proactive coping strategy adopted by students, in the effort to find alternative solutions for preserving interpersonal relationships [33,48,49,51,52]. Notably, participants in the present study did not quote loneliness as a recurrent negative emotion, rather focusing in this category on feelings of uncertainty, as well as difficulties in setting and pursuing short- and long-term educational and personal plans. Overall, they showed adequate self-regulatory strategies, turning their thoughts to the community and to their own social engagement. Concerning the latter, it was mainly reported by Health Sciences students, who were often involved as volunteers in ambulance services and emergency call centres during the pandemic. Positive thoughts and emotions were reported by students across disciplinary areas, primarily referring to hope and to the perception of the pandemic-related situation as an opportunity for personal learning and growth; these answers again suggest that participants were able to adopt proactive coping strategies, based on the appraisal of the emergency condition as an opportunity rather than a threat.

The most relevant difference emerged in participants’ narratives concerned the category *study/university*, mentioned by a significantly higher percentage of students in Humanities, who emphasized negative aspects such as inadequate organization of online activities and professors’ lack of attention and responsiveness to students’ requests and needs. Different concerns were raised by students in the Health Sciences domain, who were primarily worried about the interruption of their practical training in hospitals and outpatient services, and by the potential consequence of these restrictions on their academic and professional growth. Furthermore, they found ways to counterbalance the lack of practical training through volunteering in health services [23,53]. Consistent with our findings, previous studies have shown increased levels of psychological distress associated with students’ concerns about their academic activities [22,34], mainly due to potential delays in degree completion [23], distance learning limitations [51], and feelings of loneliness due to forced physical distance [25].

### 4.2. Resilience Levels and Students’ Experience

Participants in this study reported overall moderate to high levels of resilience. Students in the Health Sciences however reported higher mean resilience values than participants in the other two groups, with a significant difference emerging in the comparison with students in Humanities. In addition, they showed high resilience levels in higher percentage than the other groups. Previous studies highlighted that students from the Health Sciences had a more appropriate judgement and knowledge of the COVID-19 emergency [34,45,46], focusing their thoughts more on the disease itself than on the daily life consequences of the pandemic [35] and identifying themselves with their future professional role [54]. Possessing a more targeted knowledge to better interpret the health emergency and being directly involved as primary present or future actors in the pandemic management may play a role in fostering positive coping strategies and thus increasing resilience levels [47]. Gallé et al. also observed that a good level of knowledge about COVID-19 and its control (e.g., the use of masks and social distancing) may have been a protective factor during the first phase of the pandemic [33]. In apparent contrast, another study conducted in Italy during the first months of the emergency detected lower resilience levels among health professionals, compared to the general population [6]. Notably, however, resilience was negatively related to unexpected workload, awareness of the exposure to contagion in the absence of adequate personal protective equipment, and fear for infecting family members [6].

Another factor supporting resilience in our participants may be related to their living conditions. Although previous evidence was obtained about the positive association between pandemic-related isolation and affective disorders [55]; most of the participants involved in the present study were emerging adults still living with their parents and siblings. Evidence from other research supports this interpretation, showing that living in urban areas and cohabiting with parents represent protective factors against negative psychological consequences due to the COVID-19 pandemic [23]. As concerns the relationship between students’ resilience level and mention of specific categories in their narratives, only one association was detected: students with moderate levels of resilience were more likely to focus on *study/university* than students with high resilience levels, specifically highlighting problematic aspects in this domain. As documented in several studies, for university students, a major challenge during the pandemic was the change in learning mode [23,34,51]; not surprisingly, in the present study, related problems were perceived to a greater extent by less resilient participants. This finding is in line with studies that identify resilience as a psychological resource that develops through adversity [7,17,56,57]. While some of the students in our sample reported concerns and disappointment for the limitations imposed by remote academic activities, others emphasized the positive aspects of distance learning, such as the opportunity to manage their learning schedule more flexibly, the university’s positive role as a normalizing factor in times of uncertainty, and the discovery of the overall potential of distance learning.

Overall, the results of the present study confirm and extend previous research, by highlighting the interplay between resilience and adaptive coping strategies, as well as by identifying through participants’ direct narratives the intrapersonal and interpersonal protective factors mitigating the negative psychological consequences of the COVID-19 outbreak [58,59,60,61]. More specifically, highly resilient students seemed to adopt more adaptive coping strategies, such as identifying positive aspects in a negative condition, a reappraisal strategy defined as benefit finding [62].

The knowledge obtained from these findings, together with evidence derived from the increasingly vast literature on students’ experience during the pandemic, could be useful in developing more targeted support and intervention programs aimed at counterbalancing the long-term implications of the pandemic on mental health and educational careers of university students. In addition, the joint investigation of qualitative and quantitative variables provides a better understanding of the participants’ experience, and it allows for tailoring interventions according to individuals’ or groups’ needs.

Regarding mental health, as evidenced by the overall moderate to high levels of resilience, participants were aware of both the challenges posed by the pandemic, and their potential to adapt to them responsibly and proactively. These results shed light on the relevance of paying attention to the evaluation of personal and environmental resources beyond investigating pathological symptoms; researchers’ attention to individual and collective strengths can shed a more comprehensive light on the experience and self-perception of college students under ordinary and extraordinary circumstances. At the clinical level, the effectiveness of interventions can be enhanced by a careful assessment of available personal resources and areas of optimal functioning, which can thus be more effectively valued and mobilized.

Regarding educational careers, the qualitative results of the study revealed that college-based interventions should take into account the specific needs of students, according to their disciplinary area. The present study showed that the main concerns reported by Health Science students referred to the interruption of face-to-face clinical trainings, and the difficulties related to learning about applied and clinical subjects online. These findings also suggest that, even after the easing of restrictions and the retrieval of in-person activities, a variety of virtual tools and contents could be implemented and successfully incorporated in clinical internships, in line with the evolution of health care, allowing students to personalize their learning pathways and acquire new technological skills flexibly. Participants attending Political Sciences and Humanities courses were instead primarily concerned about their future academic and employment opportunities. For this reason, at least in the Italian context, the undergraduate education programs offered to these students should be implemented by including subjects related to employment negotiation and regulations, labour market opportunities, specialized job-oriented trainings, as well as a broader range of practical workshops.

### 4.3. Strengths and Limitations

The present study has strengths and limitations that should be acknowledged. As concerns strengths, it provides novel evidence of the associations between resilience and students’ experience of the first pandemic lockdown, using a mixed-method approach which allowed for gathering in-depth information of the participants’ perceived benefits and shortcomings of the situation, as well as their psychological resources. Moreover, a large sample of participants attending courses in three different disciplinary areas was involved; differences in the understanding and personal narratives of the pandemic could emerge across the curricula.

Despite these strengths, the findings of the present study should be interpreted with caution. The cross-sectional nature of the data precludes conclusions about causality. In addition, most of the participants were females; it is thus possible that the results reflect a polarization towards women’s experiences. The possibility of a self-selection bias in participation, based on personal interest and internet access/availability of online resources, should also be considered. Moreover, the study was conducted during the first outbreak of the pandemic, a peculiar phase of worldwide uncertainty and perceived vulnerability in all sectors of the society. For that reason, interpretations and applications of the findings should be carefully contextualized.

## 5. Conclusions

Although students are often considered a population at risk of experiencing distress, our findings show how psychological resources, such as resilience, can coexist and counterbalance the manifold stressors perceived during the pandemic. Moreover, qualitative findings suggested the ability of the participants to find benefits in a problematic condition, by identifying opportunities for personal and academic growth despite the pandemic related restrictions. In conclusion, results suggest that understanding both individual vulnerabilities and protective factors can help design interventions that promote students’ resilience and relieve their short- and long-term concerns, not only in times of crisis such as the pandemic. These interventions should leverage the individual’s personal strengths such as hope, meaning seeking and coping skills, factors that could be fruitfully considered when designing resilience focused training programs.

## Figures and Tables

**Table 1:** Demographic characteristics of the three groups.

	Health Sciences		Humanities		Political Sciences	
	N	%	N	%	N	%
**Gender**						
Female	224	76.19	52	76.47	39	80.41%
Male	70	23.81	16	23.53	20	19.59%
Total	294	100.00	68	100.00	59	100.00
**Degree level**						
Bachelor	196	66.67	42	61.76	41	69.49
Medical school, dentistry (6-year course)	78	26.53	0	0.00	0	0.00
Master	20	6.80	26	38.24	18	30.51
Total	294	100.00	68	100.00	59	100.00
**Employment status**						
No	216	73.47	48	70.59	36	61.02
Yes	78	26.53	20	29.41	23	38.98
Total	294	100.00	68	100.00	59	100.00
**Marital Status**						
Unmarried	269	91.50	62	91.18	54	91.53
Married	13	4.42	2	2.94	3	5.08
Cohabiting with partner	11	3.74	4	5.88	2	3.39
Divorced	1	0.34	0	0.00	0	0.00
Total	294	100.00	68	100.00	59	100.00
**Cohabitation during the pandemic**						
Alone	19	6.46	1	1.47	3	6.52
Partner	14	4.76	2	2.94	1	2.17
Partner and children	6	2.04	1	1.47	1	2.17
Partner and relatives	2	0.68	1	1.47	1	2.17
Parents	61	20.75	21	30.88	8	17.39
Siblings	4	1.36	0	0.00%	1	2.17
Parents and siblings	158	53.74	31	45.59	20	43.48
Extended family	16	5.44	4	5.88	3	6.52
Parents and Partner	0	0.00	2	2.94	1	2.17
Roommates	14	4.76	5	7.35	7	15.22
Total	294	100.00	68	100.00	59	100.00
**Religious practice**						
None	116	39.46	29	42.65	35	59.32
Occasional	97	32.99	23	33.82	13	22.03
Regular	81	27.55	16	23.53	11	18.64
Total	294	100.00	68	100.00	59	100.00

**Table 2:** Category distribution of participants’ answer units by disciplinary area.

	Health Sciences		Humanities		Political Sciences	
	N	%	N	%	N	%
**Categories**						
Work	2	0.39	0	0	1	0.89
Study/university	44	8.53	20	13.33	13	11.61
Social commitment	27	5.23	1	0.67	1	0.89
Community/society issues	189	36.63	71	47.33	53	47.32
Health management	48	9.30	5	3.33	6	5.36
Media/information	19	3.68	5	3.33	6	5.36
Family relations	17	3.29	6	4.00	1	0.89
Interpersonal relationships	15	2.91	7	4.67	4	3.57
Physical health	8	1.55	9	6.00	0	0.00
Negative thoughts/emotions	67	12.98	10	6.67	13	11.61
Positive thoughts/emotions	79	15.31	15	10.00	13	11.61
No impact	1	0.19	1	0.67	1	0.89
Total	516	100.00	150	100.00	112	100.00

**Table 3:** Distribution of the subcategories mentioned within the most frequently narrative categories across study areas.

	Health Sciences		Humanities		Political Sciences	
	N	%	N	%	N	%
**Study/university**						
Positive aspects	4	9.09	8	40.00	5	38.46
Negative aspects	44	90.91	12	60.00	8	61.54
**Community/society issues**						
Positive current aspects	54	29.19	16	22.54	10	18.87
Negative current aspects	63	34.05	29	40.85	30	56.60
Positive future expectations	55	29.73	14	19.72	9	16.98
Negative future expectations	13	7.03	12	16.90	4	7.55
**Negative thoughts/emotions**						
Anxiety/stress	16	23.88	4	26.67	2	16.67
Negative emotions	45	67.16	7	46.67	9	75.00
Negative view of the future	6	8.96	4	26.67	1	8.33
**Positive thoughts/emotions**						
Adaptive coping	10	12.66	1	8.33	1	7.69
Personal growth	37	46.84	6	50.00	8	61.54
Hope for the future	32	40.51	5	41.67	4	30.77

**Table 4:** Comparative analysis of the percentage of participants who mentioned each answer category by study area.

	Health Sciences	Humanities	Political Sciences	χ^2^
Work	0.68	0.00	1.69	1.2971 +
Study/university	10.88	23.53	13.56	7.6612 *
Social commitment	7.82	1.47	1.69	6.2019 *,+
Community/society issues	45.92	60.29	57.63	6.2121 *
Health management	13.27	5.88	8.47	3.5619
Media/information	6.12	7.35	6.78	0.1547 +
Family relations	5.44	5.88	1.69	1.5928 +
Interpersonal relationships	4.42	1.47	6.78	2.2265
Physical health	2.72	1.47	0.00	1.9117 +
Negative thoughts/emotions	16.33	19.12	18.64	0.4185
Positive thoughts/emotions	23.13	14.71	18.64	2.6069
No impact	0.34	1.47	1.69	1.9334 +
N participants ^a^	294	68	59	

^a^ Each participant could provide more than one answer; * *p* < 0.05; + at least 33% of the cells have expected counts less than 5. Chi-square may not be a valid test.

**Table 5:** Participants’ levels of resilience and mean RSA scores by disciplinary areas.

	Health Sciences			Humanities			Political Sciences		
	N	Mean	SD	N	Mean	SD	N	Mean	SD
**Levels of Resilience**									
Low	1 +	72.00		0	0.00	0.00	0	0.00	0.00
Moderate	88	136.8	14.46	36	130.1	15.80	28	138.0	13.23
High	205	173.9	11.41	32	169.3	12.76	31	173.9	14.26
RSA mean score	294	4.92	0.66	68	4.50	0.74	59	4.75	0.69

+ Not included in statistical analysis.

**Table 6:** Percentage distribution of participants mentioning each qualitative answer category by resilience level.

	Moderate Resilience	High Resilience	χ^2^
Work	0.00	1.12	1.7137
Study/university	19.08	10.07	6.8052 *
Social commitment	3.95	7.09	1.7106
Community/society issues	49.34	50.37	0.0412
Health management	11.18	11.57	0.0141
Media/information	5.26	7.09	0.5378
Family relations	7.24	3.73	2.5092
Interpersonal relationships	2.63	5.22	1.5889
Physical health	0.66	2.99	2.5050
Negative thoughts/emotions	17.76	16.42	0.1250
Positive thoughts/emotions	19.08	22.01	0.5048
No impact	0.66	0.75	0.0107

* *p* < 0.05.

## Data Availability

Data will be made available upon request to expert scientists in the field and upon clear and reasonable hypotheses.
